# Protective Effects of Quercetin on Mitochondrial Biogenesis in Experimental Traumatic Brain Injury via the Nrf2 Signaling Pathway

**DOI:** 10.1371/journal.pone.0164237

**Published:** 2016-10-25

**Authors:** Xiang Li, Handong Wang, Yongyue Gao, Liwen Li, Chao Tang, Guodao Wen, Yuan Zhou, Mengliang Zhou, Lei Mao, Youwu Fan

**Affiliations:** Department of Neurosurgery, Jinling Hospital, Medical School of Nanjing University, No. 305 East Zhongshan Road, Nanjing, Jiangsu Province, 210002, China; Uniformed Services University, UNITED STATES

## Abstract

The present investigation was carried out to elucidate a possible molecular mechanism related to the protective effect of quercetin administration against oxidative stress on various mitochondrial respiratory complex subunits with special emphasis on the role of nuclear factor erythroid 2-related factor 2 (Nrf2) in mitochondrial biogenesis. Recently, quercetin has been proved to have a protective effect against mitochondria damage after traumatic brain injury (TBI). However, its precise role and underlying mechanisms in traumatic brain injury are not yet fully understood. The aim of the present study was to investigate the effect of quercetin on the potential mechanism of these effects in a weight-drop model of TBI in male mice that were treated with quercetin or vehicle via intraperitoneal injection administrated 30 min after TBI. In this experiment, ICR mice were divided into four groups: A sham group, TBI group, TBI + vehicle group, and TBI + quercetin group. Brain samples were collected 24 h later for analysis. Quercetin treatment resulted in an upregulation of Nrf2 expression and cytochrome c, malondialdehyde (MDA) and superoxide dismutase (SOD) levels were restored by quercetin treatment. Quercetin markedly promoted the translocation of Nrf2 protein from the cytoplasm to the nucleus. These observations suggest that quercetin improves mitochondrial function in TBI models, possibly by activating the Nrf2 pathway.

## Introduction

Secondary brain damage after TBI precipitates a complex, secondary pathological process which can result in a cascade of deleterious side effects far from the site of the initial injury [1,2]. One of the predominant concerns regarding secondary injury is swelling and the release of various chemicals that promote inflammation and cell injury or even death [3,4]. Mitochondria are the main cellular source of reactive oxygen species (ROS). Their impairment increases ROS production, damaging mitochondrial proteins, DNA, and lipids, as well as disrupting cellular Ca^2+^ homeostasis, inducing apoptosis, and causing metabolic failure. Of course, the degree of mitochondrial injury or dysfunction can be an important determinant of cell survival or death spreading from mitochondrion to mitochondrion damaging the cell itself [5,6]. To this effect, mitochondrial biogenesis can enhance cellular function and promote cellular recovery from damage caused by adverse environments or pathophysiological factors. Apoptotic mechanisms must be strictly regulated to prevent neuronal death after TBI, including regulation of the mitochondrial pathway of apoptosis.

Nuclear factor erythroid 2-related factor 2 (Nrf2) is a transcription factor commonly known as the cell’s main defense mechanism against a variety of harmful stresses. Numerous studies have reported that Nrf2 plays a critical role in counteracting inflammation across a variety of experimental models [7]. The main function of Nrf2 is to activate the antioxidant response and induce the transcription of a wide range of genes that are able to counteract the harmful effects of oxidative stress, thus restoring intracellular homeostasis [8]. It has also been shown to be activated in many neurological diseases such as TBI, and is considered an endogenous compensatory adaptation against TBI [9,10]. Recent studies have demonstrated that Nrf2 plays an important role in modulating acute inflammatory responses. Under basal conditions, Nrf2 is located in cytoplasm and is bound to its inhibitor, Kelch-like ECH associated protein 1 (Keap1), which is associated with the actin cytoskeleton and prevents Nrf2 from entering the nucleus [11,12,13]. Under oxidative or xenobiotic stress conditions, Nrf2 translocates from the cytoplasm to the nucleus, resulting in a cytoprotective response which is characterized by the upregulation of a group of antioxidant enzymes and decreased sensitivity to oxidative damage.

Quercetin (3, 3′, 4′, 5, 7-pentahydroxyflavone, Que) is a flavonoid compound in fruits, vegetables, leaves, and grains. Previous experimental investigations have shown that Que has the potential to scavenge free radicals and stimulate antioxidant enzymes in various animal models [14,15]. In this study, we focused on the effects of Que-scavenging on Nrf2 after TBI. Better understanding the role of Que in Nrf2 in the context of mitochondrial biogenesis after TBI may make the interplay of nuclear and mitochondrial genomes more clear, and thus provide new insights into the molecular mechanisms of mitochondrial dysfunction. We also set out to explore whether the protective effects of Que on mitochondrial biogenesis after TBI are related to Nrf2 pathway activation. Our primary goal in conducting this study was to investigate the ameliorative effect of Que on TBI-induced mitochondria injury and the possible protective mechanisms of Que in mice.

## Materials and Methods

### Animals

Male ICR mice (Animal Experiment Center of Nanjing Medical University, Jiangsu, China) aged 6–8 weeks and weighing 28 g-32 g were used in this study. The mice were housed at 23±1°C with a 12 h light/dark cycle with free access to food and water throughout the study. Experimental protocols were approved by the Animal Care and Use Committee of Nanjing University and conformed to the Guide for the Care and Use of Laboratory Animals set by the National Institutes of Health (NIH).

### TBI Model

The TBI model used in this study is based on Marmarou’s weight-drop model as previously described by Flierl et al. and in a study we conducted previously [16,17]. Briefly, mice were anesthetized intraperitoneally with chloral hydrate and placed onto the platform directly under the weight-drop device. A midline longitudinal scalp incision of about 1.5 cm was made, then the fascia was removed to expose the skull. After locating the left anterior frontal area (1.5 mm lateral to the midline on the mid-coronal plane) as the impact area, a 200 g weight was released onto the skull. The scalp wound was then closed with standard suture material. Sham animals underwent the same procedure minus the weight drop.

### Experimental design

After 1 week of acclimation, the test mice were divided into four groups (*n* = 30 for each group): Sham group, TBI group, TBI + vehicle group, and TBI + Que group. Mice in the TBI + Que group were injected with 50 mg/kg Que intraperitoneally (Sigma Aldrich, Shanghai, China) 30 min after the onset of TBI. Mice in the TBI + vehicle group received equal volumes of vehicle (DMSO+0.9% saline) at the corresponding time points. The doses used in this study were determined based on a study on quercetin neuroprotection in an acute hypobaric hypoxia model. All the mice of each group were anesthetized and intracardially perfused with 0.9% saline followed by 4% paraformaldehyde. 24 h post-TBI.

### Tissue processing

For western blot analysis, animals were rapidly killed 24 h post-TBI and their ipsilateral cortexes collected. The tissue was positioned directly over the center of the injury site and included both contusions and penumbra. Samples were immediately frozen in liquid nitrogen and stored at -80°C until use. For immunohistochemistry analysis, the whole brain was removed 24 h after TBI and immersed in 4% paraformaldehyde overnight.

### Isolation of mitochondria

Mitochondrial and cytosolic proteins were extracted from the left cerebral cortical tissue using the Tissue Mitochondria Isolation Kit for Tissue Protocols (Beyotime Institute of Biotechnology, Shanghai, China) [18]. Fresh tissue samples were homogenized with a Polytron grinder in an ice-cold homogenization buffer and centrifuged at 1000× *g* for 5 min at 4°C to isolate the nuclear fraction. The obtained supernatants were centrifuged at 3500× *g* for 10 min at 4°C and the sediment was determined to be mitochondria. The supernatants were collected and centrifuged at 12,000× *g* for 10 min at 4°C to remove sediment and obtain cytoplasmic proteins. The brain tissue was weighed, homogenized, and centrifuged at 12,000× *g* for 15 min at 4°C, then the protein content of each sample was determined using a protein assay kit.

### Western blot analysis

Mitochondrial, nuclear, and cytosolic proteins from the cerebral cortex were extracted and quantified as described in our previous research [17,19]. After the protein concentrations were determined via Bradford method, equal protein amounts (50 ug) per lane were separated by 10% or 12% sodium dodecyl sulfate-polyacrylamide gel electrophoresis and transferred to polyvinylidene-difluoride (PVDF) membranes (Millipore, Bedford, MA, USA). Each he membrane was blocked with 5% skimmed milk for 2 hours at room temperature and incubated overnight at 4°C with primary antibodies H3 (1:1000, Cell Signaling Technology, Danvers, MA, USA), cytochrome c (1:5000, Abcam, Cambridge, MA, USA), Bax (1:400, Abcam, Cambridge, MA, USA), COX IV (1:1000, Cell Signaling Technology, Danvers, MA, USA), β-actin (1:5000, Bioworld Technology, MN, USA), and Nrf2 (1:1000, Abcam, Cambridge, MA, USA). After being washed with TBST (3×10min), the membranes were incubated with goat anti-rabbit horseradish peroxidase (HRP)-conjugated IgG (1:5000, Bioworld Technology, St. Louis Park, MN, USA) for 2 h at room temperature. The membranes were then incubated for 2 h with corresponding secondary antibodies. The protein bands were visualized by enhanced chemiluminescence (ECL) Western blot detection reagents (Millipore, Billerica, MA, USA) and exposure to X-ray film. Developed films were digitized on an Epson Perfection 2480 scanner (Seiko Corp., Nagano, Japan). Band density was quantified using Un-Scan-It 6.1 software (Silk Scientific Inc., Orem, UT, USA), and data were normalized to COX IV, _β-actin, or Histone 3.

### Immunohistochemical staining

Brain tissue samples were fixed in formalin for 24 h and embedded in paraffin. For immunohistochemical examination, the tissue sections (4 mm) were incubated with an Nrf2 (1:100, Abcam, Cambridge, MA, USA) overnight at 4°C, followed by a 15 min wash in phosphate-buffered saline (PBS). The sections were then incubated with horseradish peroxidase-conjugated IgG (1:400, Santa Cruz Biotechnology, Santa Cruz, CA) for 1 h. Immunolabelling was visualized as brown using diaminobenzidine, with haematoxylin counterstaining. Immunohistochemical-positive neurons were counted by two independent investigators who were kept from knowing the injured group from the sham group. Six fields were selected randomly for each section and observed under a light microscope (ECLIPSE E100, Nikon, Japan), where the mean number of positive neurons in six views was regarded as the data for each sample.

### Mitochondrial SOD and malondialdehyde (MDA) content

Mitochondrial MDA content and SOD activity were measured using a spectrophotometer according to the manufacturer’s instructions (Nanjing Jiancheng, Nanjing, China). Total protein concentrations were determined via Bradford method [20,21,22]. MDA content was expressed as nmol/mg protein and SOD activity was expressed as U/mg protein.

### Mitochondrial membrane potential (MMP) measurement

Changes in the mitochondrial membrane potential (MMP) were analyzed using 5,59,6,69-tetrachloro-1,193,39-tetraethylbenzimidazole carbocyanine iodide (JC-1; Molecular Probes, Inc., Eugene, OR, U.S.) [23]. The fluorescence intensity was recorded on a flow cytometer (FACScan; BD Biosciences) and analyzed in WinMDI 2.9 software. The emitted orange and green fluorescence was collected through 585/42 nm (FL2) and 530/30 nm (FL1) bandpass filters. The FL1–FL2 compensation was 4% and the FL2–FL1 compensation was 44%. Bivariate plots of FL2 versus FL1 were utilized to analyze MMP.

### Intracellular ATP content measurement

The intracellular ATP level was measured as previously described with a Bioluminescent Somatic Cell Assay Kit (Sigma-Aldrich) [24]. Viable mitochondria were lysed to release the intracellular ATP, mixed with substrate and luciferase enzyme, and transferred into a 96-black well plate for luminescence analysis with an OrionL Microplate Luminometer (Berthold, Bad Wildbad, Germany).

### Statistical analysis

Data are expressed as mean±SEM as determined by Tukey’s post hoc tests. Statistical significance was considered *p* < 0.05. Analyses were performed in SPSS 17.0 (SPSS Inc., Chicago, IL, USA).

## Results

### Western blot analysis

As discussed in the Introduction, Que is known to significantly increase the activities of antioxidant enzymes following TBI though the underlying molecular mechanisms remain elusive. Nrf2 is known to play an important role in the activation of antioxidant enzymes, so it is reasonable to hypothesize that Que might activate Nrf2 thus enhancing antioxidant enzyme activity. The well-established, classic activation pattern of Nrf2 is translocation from the cytoplasm to the nucleus.

**Fig 1:**
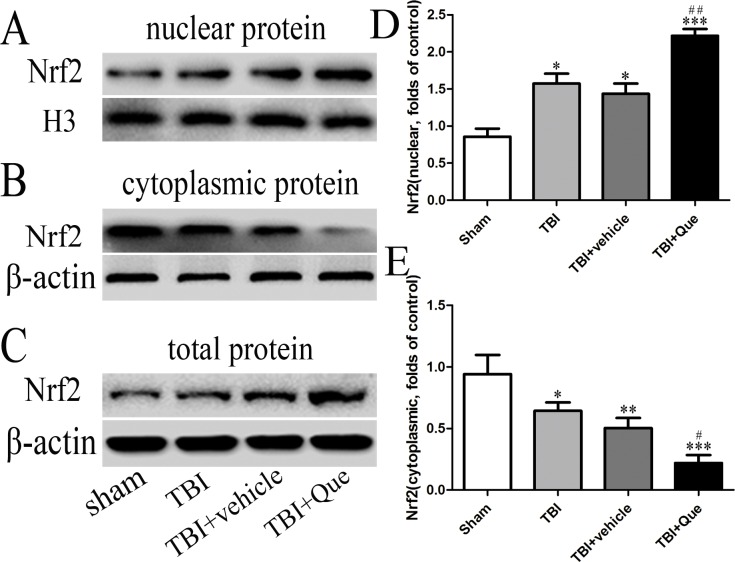
Que promoted translocation of Nrf2 from cytoplasm to nucleus and enhanced Nrf2-ARE binding. (A) The nuclear protein Nrf2 expression after Que treatment in mice with TBI, as measured by western blot. (B) The cytoplasmic protein Nrf2 expression after Que treatment in mice with TBI, as measured by western blot. (C) The total protein Nrf2 expression after Que treatment in mice with TBI, as measured by western blot. (D-E) The ratios of cytoplasmic Nrf2 and nuclear Nrf2. Bars represent the mean ± SEM (n = 5 per group). *P<0.05, **P<0.01 and ***P<0.001 compared with the sham group; ^#^P<0.05 and ^##^P<0.01 compared with the TBI + vehicle group.

Our results showed that compared to the sham group, both TBI and Que were indeed inducers of Nrf2 nuclear translocation (Fig 1A, 1D and 1E). In addition, compared to the vehicle-treated group, the Que-treated group had a significantly increased ratio of nuclear Nrf2 and a reduced ratio of cytoplasmic Nrf2 (Fig 1A, 1B, 1D and 1E), suggesting that Que promoted Nrf2 nuclear translocation (Fig 1A, 1B and 1C). These observations indicate that Que increases the expression of total Nrf2 after TBI (Fig 1C).

**Fig 2:**
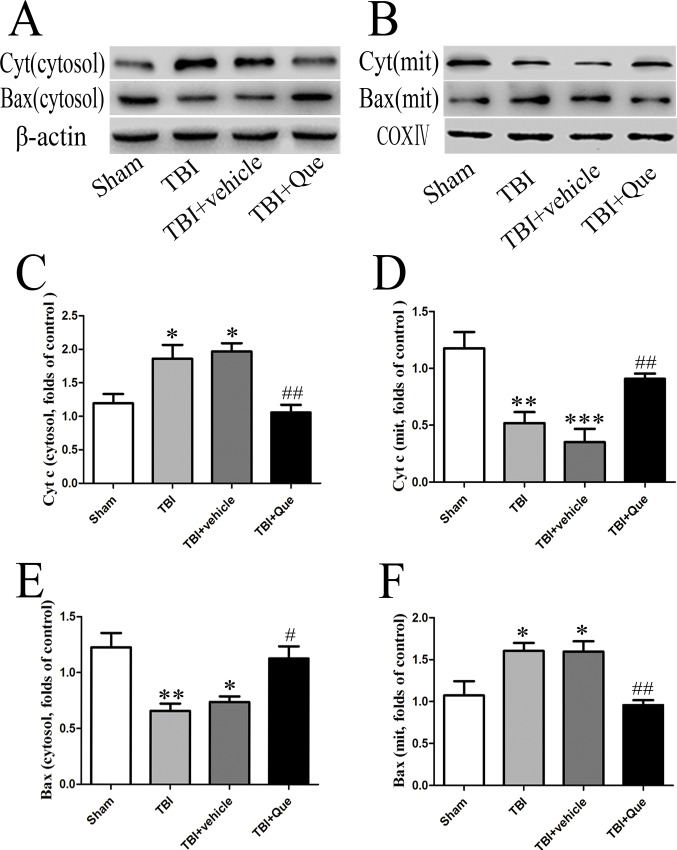
Effect of Que on pro-apoptotic protein expression following TBI. **(A, B) The expression of Bax and cytochrome c in the ipsilateral cortex was evaluated by Western blotting 24 h after injury.** Representative blots show the relative expression of (D, F) mitochondrial and (C, E) cytosolic Bax and cytochrome c. Expression was normalized to the level of β-actin or COX IV. Data represent the mean ± SEM (n = 5 per group). *P<0.05, **P<0.01 and ***P<0.001 compared with the sham group; ^#^P<0.05 and ^##^P< 0.01 compared with the TBI + vehicle group.

In addition, the levels of Bax protein in mitochondria and cytosol increased and decreased after TBI, respectively, as compared to the sham and sham + vehicle groups (Fig 2B, 2D and 2F) while mitochondrial and cytosolic cytochrome c levels decreased and increased, respectively, relative to sham animals (Fig 2A, 2C and 2E). These effects were reversed in TBI mice treated with quercetin, in which mitochondrial translocation of Bax and subsequent cytosolic cytochrome c release were inhibited.

### Immunohistochemical staining

**Fig 3:**
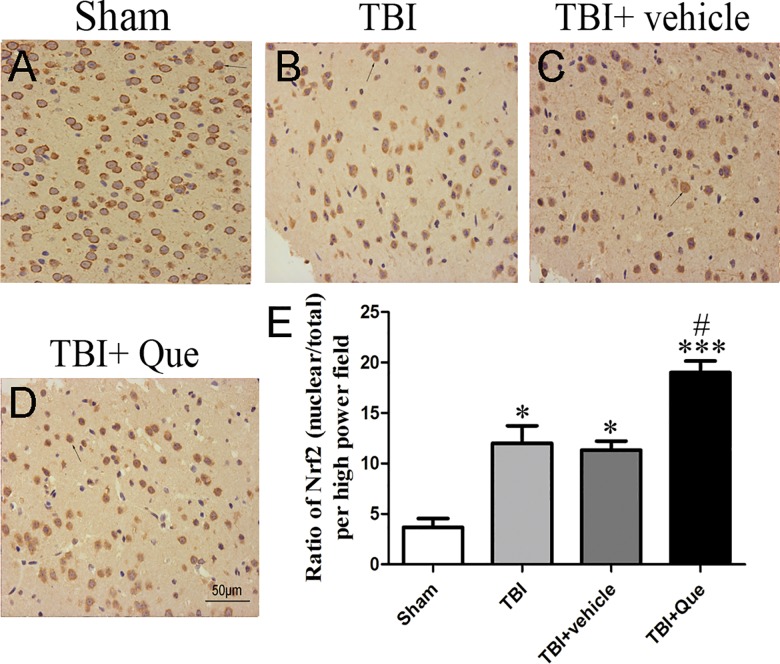
The representative photomicrographs showing Nrf2 immunohistochemistry of tissue from different group 24h after TBI. As compare with sham group, TBI group presented a morphology with Nrf2 concentrated in the nucleus, and after treating with Que, this concentration morphology was more apparent. Data represent the mean ± SEM (n = 5 per group). *P<0.05 and ***P<0.001 compared with the sham group; ^#^P<0.05 compared with the TBI + vehicle group. Scale bar: 20 μm.

As shown in (Fig 3), TBI enhanced Nrf2 expression in the nucleus and quercetin enhanced Nrf2 concentration in the nucleus. These may indicate that Que promotes Nrf2 translocation from cytoplasm to nucleus and thereby lends elevated binding ability to the downstream genes. Relatively few Nrf2 immunostained neurons were observed in the sham samples of the cerebral cortex. In the TBI group and TBI + vehicle group, conversely, the number of Nrf2-positive neurons increased markedly compared to sham group (Fig 3B and 3C) but decreased in the TBI + quercetin group (Fig 3D).

### Mitochondrial SOD and MDA content

**Fig 4:**
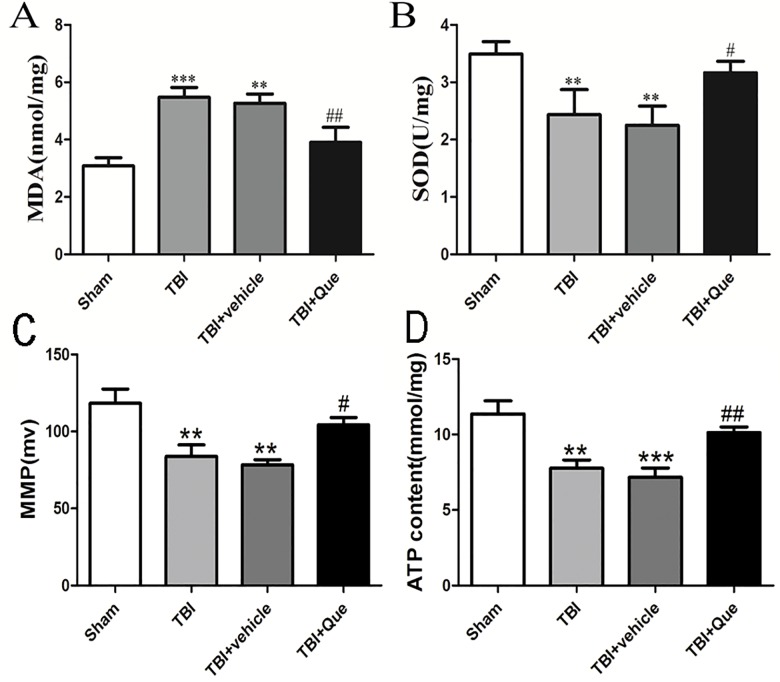
Que attenuated Mitochondrial oxidative stress and biogenesis caused by TBI. (A) Measurements of MDA levels. (B) The activities of SOD. (C) MMP. (D) Intracellular ATP content. Data represent the mean ± SEM (n = 5 per group). **P<0.01 and ***P<0.001 compared with the sham group; ^#^P<0.05 and ^##^P<0.01 compared with the TBI + vehicle group.

Elevated mitochondrial MDA was detected in the TBI and TBI + vehicle groups (Fig 4A). Administration of Que significantly reduced the generation of mitochondrial MDA. Mitochondrial SOD decreased after TBI (Fig 4B), but Que significantly upregulated the SOD level.

### Mitochondrial membrane potential (MMP) and Intracellular ATP content

The MMP and Intracellular ATP content values reduced in the TBI and TBI + vehicle groups, and increased significantly following treatment with quercetin(Fig 4C and 4D).

## Discussion

Que is a highly effective antioxidant that has been frequently used as a protectant in TBI studies. Que is also a drug which can increase mitochondrial electron transport activity while stimulating a broad anti-ROS program, which makes it an almost ideal protein for limiting the damage associated with defective mitochondrial function seen in many neurodegenerative diseases [25,26]. However, alongside the growing evidence for mitochondrial participation in traumatic neuronal injury, new neuroprotective approaches must include strategies aimed toward limiting and reversing mitochondrial dysfunction [27,28].

This information inspired us to further explore the inner mechanism by which Que protects the brain’s mitochondrial biogenesis from oxidative injury. A number of studies have shown that Que protects the mitochondrial structure and influences mitochondrial structure, function, and biogenesis [29,30]. The exact mechanisms of these effects are unknown, but are likely multifaceted and related to mitochondrial response to the Nrf2 signaling pathway after TBI. To the best of our knowledge, this is the first study to evaluate the effects of Que on mitochondrial function modulation in a TBI model. We found that treatment with Que after TBI attenuated brain mitochondrial biogenesis after contusion-induced oxidative injury. The increased mitochondrial MDA content and decreased SOD activity in the cortex of injured mice indicated that oxidative stress indeed occurred after TBI.

The dose of Que (50 mg/kg, i.p.) we used was determined based on a previous TBI study [31,32,33]. Many recent studies have demonstrated the importance of the therapeutic time-window after the initial TBI [19,34]. It is critical to begin intervention as soon as possible following TBI to achieve maximum neuroprotection. The protective effects of Que via its anti-oxidative characteristics have been demonstrated in many TBI studies [35,36,37]. Our results also indicated increased expression of Nrf2-related transcription factors associated with mitochondrial biogenesis after Que treatment. Similarly, the mitochondrial respiratory chain consisting of four membrane-bound complexes (COXⅠ–Ⅳ) is known to be involved in ATP synthesis [38,39]; in addition, increase in cytochrome c concentration typically occurs in conjunction with increases in other mitochondrial enzymes of the electron transport chain and enzymes in the tricarboxylic acid cycle. These changes altogether lead to an overall increase in mitochondrial capacity [28,40]. In short, previous reports of Que-induced increases in mitochondrial biogenesis are consistent with the increases in cytochrome c protein expression observed in our study.

The underlying molecular mechanisms related to Que in TBI remain undetermined. In effort to confirm that the Nrf2 signaling pathway is involved in the mitochondrial protection role of Que, we investigated changes in the Nrf2 signaling pathway after Que treatment. We observed Nrf2 translocation from the cytoplasm to the nucleus after TBI and found that Que further promoted this translocation. This observation is consistent with the classic pattern of Nrf2 activation in protein expression. Cytoplasmic Nrf2 protein was significantly down-regulated after TBI and was even lower after Que was administered; moreover, the total protein of Nrf2 did not change significantly after Que treatment. These findings demonstrate that rather than increasing protein expression, the influence of Que on Nrf2 is inducing its translocation from the cytoplasm to the nucleus. We would infer, accordingly, that Que regulates Nrf2 at the post-translation level (at least, in the model we used here) rather than through transcription or translation. This finding is in agreement with former studies that revealed similar mechanisms of Nrf2 regulation post-translation.

## Conclusion

In this study, we found that Que attenuated the oxidative injury to mitochondria by enhancing the expression and activity of antioxidant enzymes in a TBI model. We also found that the administration of Que resulted in the activation of the Nrf2 pathway. The effects of Que in preventing the decline of antioxidant enzyme activity on mitochondrial biogenesis after TBI may be attributed to its influence on Nrf2 pathway modulation.

## Supporting Information

S1 Fig Translocation of Nrf2 from cytoplasm to nucleus. (RAR)

S2 Fig Pro-apoptotic protein expression in cytoplasm to mitochondria. (RAR)

S3 Fig Date of immunohistochemistry. (RAR)

S1 File Dataset. (RAR)
